# Noradrenergic Modulation of Light-Driven Egr-1 Expression in the Adult Visual Cortex

**DOI:** 10.4137/jen.s6497

**Published:** 2011-03-03

**Authors:** Liisa A. Tremere

**Affiliations:** Department of Brain and Cognitive Sciences, University of Rochester, Rochester, NY 14627, USA. Department of Geriatric Medicine and ROCA University of Oklahoma Health Sciences Center Oklahoma City, OK 73104-0505

**Keywords:** noradrenaline, visual cortex, V1, plasticity

## Abstract

Noradrenaline has been shown to modulate sensory driven responses in the primary visual cortex (V1) of a number of vertebrate species. Moreover, this neurotransmitter has been postulated to bridge neuronal activation to genomic responses in order to instruct cells in long-lasting changes in neuronal performance. Here we show that local noradrenergic receptor activation in V1 is required for experience-regulated gene expression in the mouse V1. More specifically, we demonstrate that noradrenaline used locally within V1 mediates the light-driven gene expression of egr-1, an immediate early gene implicated as a mediator of neuronal plasticity. Visually-driven egr-1 expression largely depends on the α-adrenergic receptor subtype, with a lesser involvement of the β-subtype. Our findings suggest that noradrenergic transmission regulates plasticity associated gene expression in V1 of awake mice and is well positioned to broadly integrate experience-dependent changes at the cell's membrane and the genomic machinery in neurons.

## Introduction

Noradrenergic transmission plays a central role in regulating attentional processing, sensory learning and the consolidation and recall of behaviorally-relevant memories.^1–4^ Within the rodent primary visual cortex (V1), noradrenaline was shown to modulate neuronal firing properties^5^ and various forms of experience-dependent synaptic plasticity.^6–9^ Importantly, ablation of the projections from the locus coeruleus, the primary source of noradrenaline to the brain, was shown to significantly suppress experience-driven gene expression in V1.^10^ Together, these data suggest that noradrenergic input modulates neuronal responses in V1 and may play a key role in coupling neuronal activation to genomic responses in visual cortical neurons, the latter of which are thought to underlie long-lasting changes in neuronal performance and, ultimately, forms of sensory learning.

In the present work we explored this hypothesis by investigating whether local noradrenergic receptor activation in V1 is necessary for experience-regulated gene expression in the mouse V1. Previous work has shown that visual experience drives a robust and transient expression of activity-dependent genes such as egr-1 and *arc* in the V1 of freely-behaving rodents.^10–12^ Interestingly, the strength of the activation of these experience-dependent genes is significantly more robust in paradigms known to induce anatomical and functional plasticity in V1 circuitry.^10,13,14^ A causal relationship between noradrenergic modulation and light-induced expression of plasticity-associated genes, however, remains to be determined. Here we explored whether local noradrenergic transmission in V1 is required for light-driven gene expression of Egr-1. Using local pharmacological approaches in the awake, restrained mouse, we show that noradrenergic transmission is central for light-induced regulation of egr-1 in V1. More specifically, experience-regulated egr-1 expression is largely dependent on α-adrenergic receptors and, to a lesser extent, on receptors of the β-subtype. Together, our findings suggest that egr-1 expression in V1 requires noradrenergic transmission in V1 of awake mice. Our data also suggest that noradrenaline is well positioned to integrate experience-dependent changes in electrophysiological properties to the genomic machinery in V1 neurons.

## Material and Methods

### Animals, head-post implants and restraint adaptation

A total of 14 female Swiss Webster mice were used in this study. Animal protocols were approved by the University of Rochester Committee on Animal Resources and are in accordance with NIH guidelines. Animals were anesthetized with Equithesin (sodium pentobarbital (50 mg/kg) and chloral hydrate (200 mg/kg)) and placed in a stereotaxic device. Two small holes were drilled over the target area in V1 (AP −2.8; ML ± 2.5; DV 0.4 mm), and dental cement was used to form a well around the injection sites and to attach a metal head post to the skull. After a recovery period of a minimum of 48 hours, animals were subjected to a restraint adaptation protocol in the dark. Each restraint session consisted of gentle immobilization of the animal for 10 minutes, in the presence of the investigator, in a body tube and attachment of the head-post to an adaptor in the stereotaxic device. These restraint sessions were repeated every 2 hours, over a 10-hour period. Following the restraining period, animals were returned to their home cages, which were also maintained in complete darkness.

### In-vivo injections in the awake mouse

**Figure 1:**
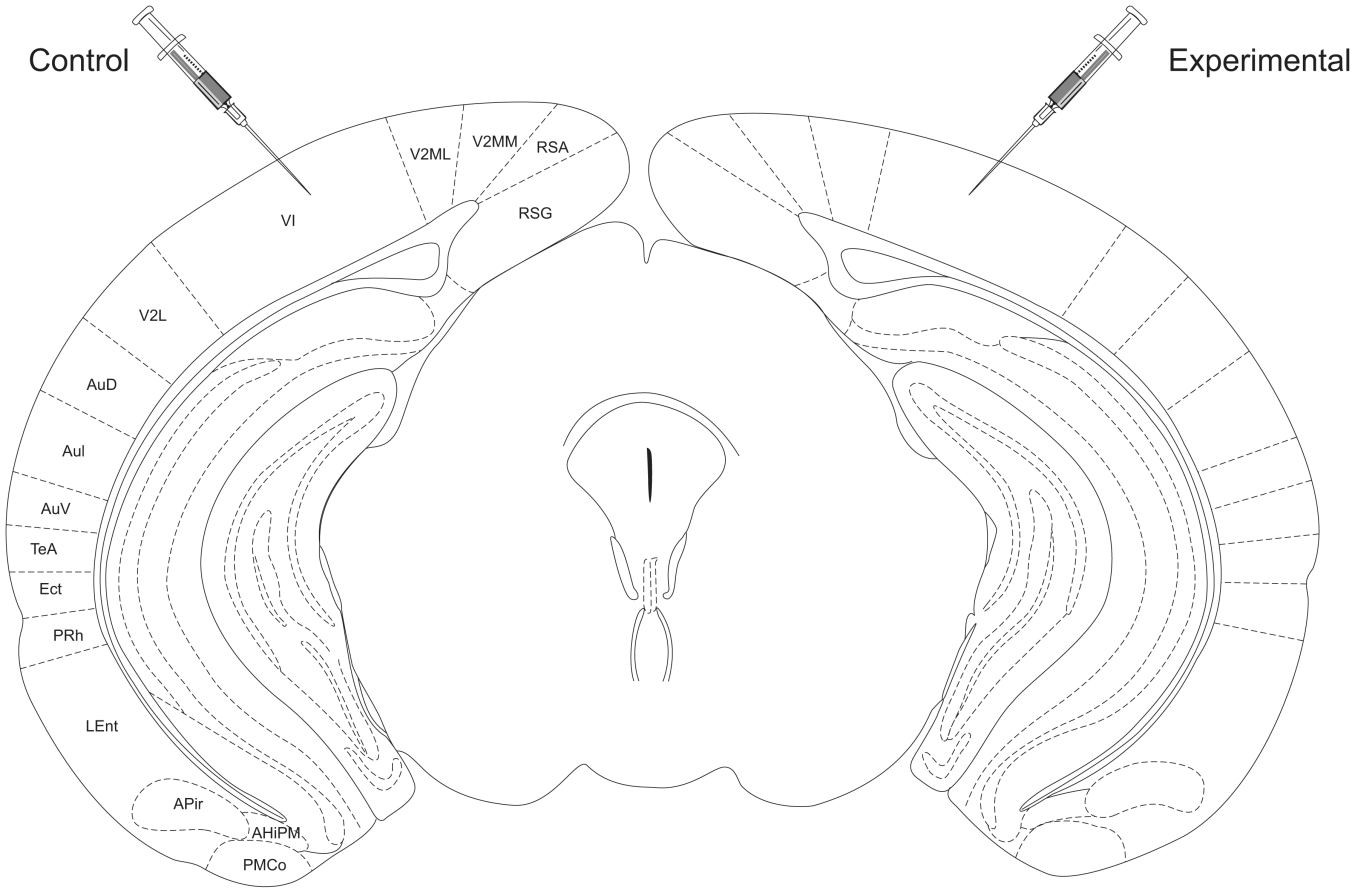
Schematic representation of a coronal section through the mouse brain (AP −2.8 mm), indicating the location of bilateral intra-cerebral injections. This stereotaxic level was used for all in-vivo injections carried out in this study.

After adaptation sessions, mice were restrained, as described above, for unilateral intracerebral injections of antagonists propranolol (300 mM in 200 μl) or phentolamine (200 mM in 200 μl). Vehicle was always injected contralaterally (Fig. 1). Injections were carried out with small glass pipettes and solutions were infused slowly (over 2 minutes) via a calibrated hydraulic pump. After injections, animals were returned to their home cages and stimulated with ambient light for 1 hour. Mice were then infused with an overdose of Equithesin and perfused transcardially with 20 ml of 0.1M PBS (pH 7.4) and a cold solution of 4% paraformaldehyde. Brains were then extracted, cryoprotected in a 30% sucrose solution, embedded in Tissue-Tek, fast-frozen in a dry-ice/ethanol bath and cut at 20 μm on a cryostat for histological processing.

### Immunocytochemistry (ICC)

Egr-1 protein expression was revealed by standard immunocytochemical procedures, as described previously (Pinaud et al, 2000). Briefly, sections containing V1 were sequentially incubated in 1) blocking buffer (0.5% albumin, 0.3% Triton in 0.1M PB) for 30 min at room temperature; 2) anti-egr-1 primary antibody, raised in rabbit, in BB overnight at 4 °C (dil 1:1000; Santa Cruz Biotechnology, USA); 3) biotinylated goat-anti-rabbit IgG, in BB, for 2 hr at RT (dil 1:200; Vector Labs, USA); 4) avidin-biotin solution in dH_2_O, for 2 hr at RT (dil 1:100; Vector Labs, USA). Each step detailed above was separated by 3 × 10 min washes in PB. Sections were subsequently developed by incubation in a solution containing 0.03% diaminobenzidine, 0.15% nickel ammonium sulfate in PB, to which 0.001% hydrogen peroxide had been added.

### Quantification of Egr-1 expression and statistical analyses

Grids of 100 × 100 μm were placed either in the supragranular, granular and infragranular layers of V1, and egr-1 positive neurons were counted as profiles under brightfield microscopy and averaged across animals for each treatment. A one-way ANOVA tested for significant effects of drug treatment with an alpha value set for *P* < 0.05.

## Results

### Egr-1 expression is regulated by visual experience in V1

**Figure 2:**
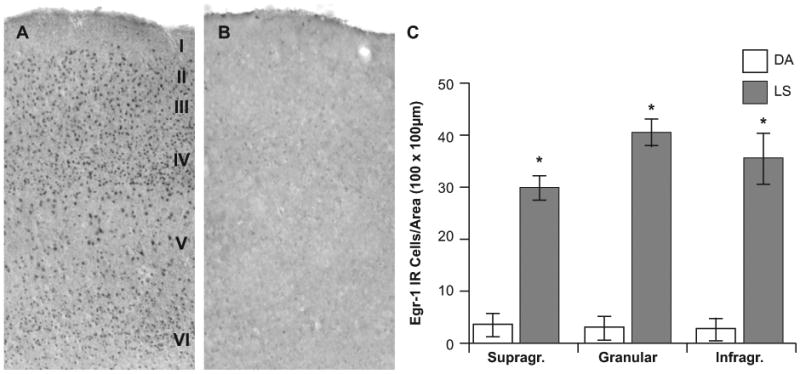
Light stimulation drives *egr-1* expression in V1. **A**) Light stimulation following a dark adaption period triggered a significant induction of egr-1 in neurons across all layers of V1, with the exception of layer I. **B**) A significant suppression of egr-1 expression levels was detected in the V1 of dark adapted animals. **C**) Light stimulation led to an average 8-fold increase in the number of light-driven Egr-1-positive neurons; this increase was most prominent in the granular layer, although also significantly marked for supra- and infragranular layers of V1. Scale bar = 100 μm.

To determine the extent to which Egr-1 is regulated by visual experience, we dark-reared a group of mice, individually, overnight. The following day, a subset of animals were either killed in the dark, or exposed to 1 hour of ambient light stimulation. Consistent with previous findings from other groups, we found that few Egr-1-positive cells could be detected in the V1 of dark-adapted animals (Fig. 2). In contrast, we observed a marked upregulation of Egr-1 protein in the V1 of light-stimulated animals. Egr-1-positive neurons could be detected across all cortical layers with the exception of layer I. To quantify this effect and determine the extent to which light stimulation differentially affect the expression of Egr-1, we quantified immunopositive neurons in the supragranular (II/III), granular (IV) and infragranular (V/VI) layers separately. In dark-adapted animals, we found that 4.1 ± 2.3, 3.2 ± 2.2 and 3.0 ± 2.6 Egr-1-positive cells could be detected in the supragranular, granular and infragranular layers, respectively. Light-stimulation led to a significant increase in the number of immunolabeled neurons across V1 cortical layers. Specifically, we found that 31.2 ± 2.1, 42.1 ± 2.5 and 35.9 ± 4.4 Egr-1 positive cells were found in the supragranular, granular and infragranular layers of V1, respectively. This difference amounted to a 8-fold increase in the number of immunopositive cells as a result of visual experience. These data indicate that dark adaptation suppresses Egr-1 expression levels, and that light stimulation drives a significant upregulation of this transcription factor in V1 neurons.

### Blockade of β-adrenergic receptors partially suppresses light-induced Egr-1 expression

To determine the extent to which the local actions of noradrenergic input regulate the light-induced expression of Egr-1, we carried out bilateral, in-vivo pharmacological injections within the V1 of awake, restrained mice. Importantly, vehicle was injected unilaterally and noradrenergic antagonists were injected contralaterally; thus, each of our animals served as its own control.

**Figure 3:**
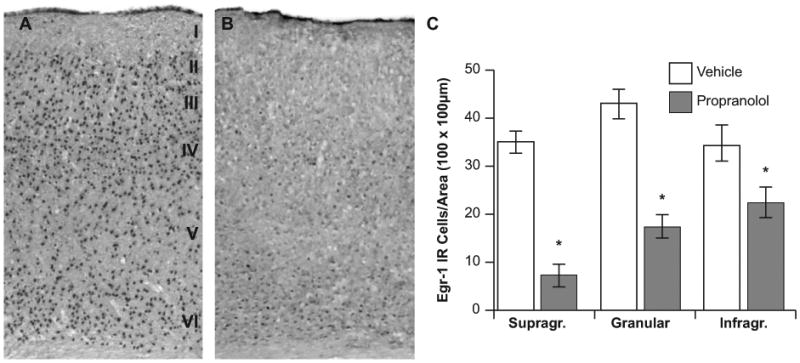
Blockade of β-adrenergic receptors partially suppress light-induced *egr-1* expression. **A**) Intra-cerebral injection of vehicle did not interfere with the pattern of light-induced egr-1 expression in V1. **B**) A single infusion of propranolol, a β-adrenergic antagonist, significantly decreased the number of egr-1 immunoreactive neurons across all layers of V1. **C**) Propranolol treatment differentially decreased the number of immunolabeled neurons across different V1 layers: the most pronounced suppression of egr-1 expression levels was detected in the supragranular layers (78%), followed by the granular and infragranular layers (59% and 35%, respectively). Scale bar = 100 μm.

Vehicle-injected hemispheres of light-stimulated animals underwent a marked light-induced upregulation of Egr-1 across all cortical layers. Layer-specific quantitative analyses revealed that 35.0 ± 1.9, 43.1 ± 1.8 and 34.6 ± 2.2 (mean ± S.E.) Egr-1-positive cells were detected in the supragranular, granular and infragranular layers, respectively (Fig. 3). Importantly, these cell densities were not statistically different from non-injected animals (*P* = 0.18). These findings suggest that vehicle injections do not impact the light-induced expression of Egr-1 in V1. In contrast, contralateral infusion of propranolol, a selective β-adrenergic antagonist, significantly decreased the number of immunolabeled neurons in V1. More specifically, we found that propranolol-infused hemispheres exhibited 7.7 ± 1.9, 17.6 ± 2.1 and 22.7 ± 1.6 Egr-1-positive neurons in supragranular, granular and infragranular layers of V1, which reflected significant decreases of 78%, 59% and 35% in the number of Egr-1 immunolabeled neurons (all *P* < 0.05). These data indicate that activation of β-adrenergic receptors in the V1 of awake, restrained mice is partially required for the light-induced expression of Egr-1.

### Blockade of α-adrenergic receptors suppresses light-induced Egr-1 expression

We next assessed whether α-adrenergic receptors also contribute to the light-induced expression of Egr-1. As with the experiment above, bilateral, in-vivo pharmacological infusions were carried out in the V1 of awake, restrained mice (vehicle was infused unilaterally, and phentolamine, a selective α-adrenergic receptor antagonist, was infused contralaterally).

**Figure 4:**
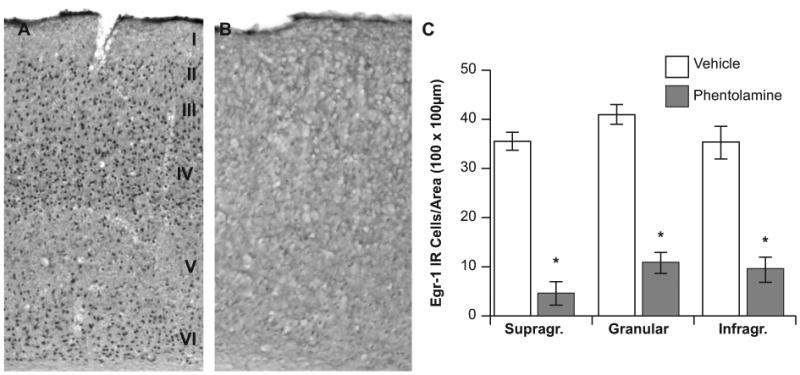
Blockade of α-adrenergic receptors largely suppresses light-induced *egr-1* expression. Light-induced egr-1 expression was not affected by vehicle infusion **A**) but was markedly suppressed by a single local injection of phentolamine, an a-adrenergic receptor antagonist **B**). **C**) Phentolamine almost completely blocked light-induced egr-1 expression in V1. An average 79% decrease in the number of egr-1 immunolabeled neurons was detected in the experimental hemisphere, compared to the vehicle-injected hemisphere. Phentolamine treatment equally impacted all layers of V1, and yielded a suppression of 88%, 74% and 74% in supragranular, granular and infragranular layers, respectively. Scale bar = 100 μm.

We found that vehicle-injected hemispheres displayed a significant light-induced upregulation of Egr-1 across all cortical layers. In particular, we found 35.7 ± 1.5, 41.3 ± 1.2 and 35.7 ± 1.7 Egr-1-positive cells in the supragranular, granular and infragranular layers of V1, respectively; these cell densities did not differ from non-injected animals (*P* = 0.09). Remarkably, we found that contralateral infusion of phentolamine largely abolished light-induced Egr-1 expression in V1 (Fig. 4). Specifically, phentolamine-injected hemispheres displayed 4.3 ± 2.2, 10.6 ± 1.8 and 9.3 ± 1.6 Egr-1-positive cells in supragranular, granular and infragranular layers of V1, which reflected significant decreases of 88%, 74% and 74% in Egr-1 immunopositive neuronal density (all *P* < 0.05). These findings directly demonstrate that α-adrenergic receptor activation in the V1 of awake mice are essential for the light-induced expression of Egr-1.

## Discussion

Here we show that local noradrenergic transmission is required for the expression of *egr-1*, an immediate early gene that has been repeatedly implicated in synaptic plasticity. More specifically, we show that blockade of noradrenergic receptors in V1 of awake mice largely abolishes the light-induced expression of egr-1 across all cortical layers, with the exception of layer I. We also found that whereas β-adrenergic receptors partially regulate the expression of this gene, blockade of α-adrenergic receptors virtually abolishes egr-1 expression in V1 neurons. These discoveries build upon previous work demonstrating that noradrenergic input regulates basal expression of egr-1 in the neocortex^15^ and that ablation of locus coeruleus neurons disrupts light induced egr-1 expression of other experimental models.^10^

Our findings also demonstrate that decreases in the number of light-induced egr-1 positive cells mediated by either α- or β-adrenergic receptor antagonism is more pronounced for the supragranular layers of V1. Based on the well known connectivity of V1, our findings suggest that noradrenergic transmission regulates plasticity-associated gene expression in neurons primarily engaged in intra-cortical connectivity. These findings are also congruent with the higher density of both α- and β-adrenergic receptor expression described for the mouse V1. In primary sensory cortices, including V1, neurons in the supragranular layers undergo marked levels of activity dependent plasticity, including synaptically-evoked long-term potentiation and depression.^16^ In fact, noradrenergic transmission has recently been shown to act as a neurochemical “switch”, controlling both the polarity of synaptic plasticity changes in V1^9^ and, based on the present findings, the engagement of molecular scripts putatively involved in experience-dependent neural rewiring.

Egr-1 expression has also been shown to require NMDA activation, a glutamatergic receptor subtype that carries a robust calcium conductance and that has been repeatedly implicated in synaptic plasticity, learning and memory formation. Notably, noradrenergic activation that is mediated by α-adrenergic receptors also controls the mobilization of intracellular calcium stores. Given the dependence of egr-1 on calcium for its expression, and the necessity of noradrenergic transmission for the expression of this immediate early gene, it is plausible that calcium regulation, either via NMDA or noradrenergic receptor activation (or a combination of both) may be a point of convergence in the intracellular cascades mediating egr-1 expression and the initiation of neuroplasticity mediated changes in sensory cortex. Different adrenergic receptor subtypes (α versus β) exert disparate effects on the cellular biology of neurons. For example, whereas α-adrenergic receptors are coupled to stimulatory g-proteins, receptors of the β-subtype engage inhibitory g-proteins. Our findings indicate that α-adrenergic receptors contribute the brunt of egr-1's light-induced regulation; consequently, these observations indicate that enhancement of adenylyl cyclase activity putatively accounts for the induction of egr-1 in V1. Given however, that antagonism of α-receptors also partially inhibited the visually-driven *egr-1* response, the regulation of this gene may involve complex and/or alternative biochemical pathways. In addition, *egr-1* expression may be modulated by different receptor subtypes in neurochemically-distinct cell types (eg, excitatory versus inhibitory neurons). Future work will shed light on these issues, as well as whether or not different types of α- or β-receptor subtypes (eg, α1, α2, β1) are required for differential regulation of egr-1 in V1 neurons.

In summary, we show that noradrenergic modulation of V1 neurons is required for the light-induced expression of the plasticity-associated gene *egr-1*. Given the known roles for noradrenaline in the gating of visual attention, this neurotransmitter may be well positioned to enhance the neural representation of behaviorally-relevant visual cues and consequently consolidate sensory memories of these events, a process that may require egr-1 induction. This possibility should be the focus of future research.
